# Expert consensus on maintenance treatment for metastatic colorectal cancer in China

**DOI:** 10.1186/s40880-015-0067-x

**Published:** 2016-01-14

**Authors:** Rui-Hua Xu, Lin Shen, Jin Li, Jian-Ming Xu, Feng Bi, Yi Ba, Li Bai, Yong-Qian Shu, Tian-Shu Liu, Yu-Hong Li, Chun-Mei Bai, Xiang-Lin Yuan, Jun Zhang, Gong Chen, Ai-Ping Zhou, Ying Yuan, Xi-Jing Wang, Xiao-Ping Qian, Yan-Hong Deng

**Affiliations:** Sun Yat-sen University Cancer Center; State Key Laboratory of Oncology in South China, Collaborative Innovation Center of Cancer Medicine, Guangzhou, 510060 Guangdong P. R. China; Department of Medical Oncology, Sun Yat-sen University Cancer Center, 651 Dongfeng Road East, Guangzhou, 510060 Guangdong P. R. China; Department of Gastrointestinal Medical Oncology, Peking University Cancer Hospital, Beijing, 100142 P. R. China; Department of Oncology, Tongji University Tianyou Hospital, Shanghai, 200331 P. R. China; Department of Medical Oncology, The 307th Hospital of Chinese People’s Liberation Army, Beijing, 100071 P. R. China; Department of Medical Oncology, West China Hospital of Sichuan University, Chengdu, 610041 Sichuan P. R. China; Department of Gastrointestinal Medical Oncology, Tianjin Medical University Cancer Institute and Hospital, Tianjin, 300060 P. R. China; Department of Medical Oncology, The 301th Hospital of Chinese People’s Liberation Army, Beijing, 100853 P. R. China; Department of Medical Oncology, Jiangsu Provincial Hospital, Nanjing, 210029 Jiangsu P. R. China; Department of Medical Oncology, Fudan University Zhongshan Hospital, Shanghai, 200032 P. R. China; Department of Medical Oncology, Peking Union Medical College Hospital, Beijing, 100032 P. R. China; Department of Medical Oncology, Tongji Hospital of Tongji Medical College, Huazhong University of Science and Technology, Wuhan, 430030 Hubei P. R. China; Department of Oncology, Rui Jin Hospital, Shanghai Jiao Tong University School of Medicine, Shanghai, 200035 P. R. China; Department of Colorectal Surgery, Sun Yat-sen University Cancer Center, Guangzhou, 510060 Guangdong P. R. China; Department of Medical Oncology, Cancer Hospital Chinese Academy of Medical Sciences, Beijing, 100021 P. R. China; Department of Medical Oncology, The Second Hospital of Zhejiang University School of Medicine, Hangzhou, 310009 Zhejiang P. R. China; Department of Oncology, The Second Affiliated Hospital of Xi’an Jiaotong University, Xi’an, 710004 Shanxi P. R. China; Department of Medical Oncology, Nanjing Drum Tower Hospital, Nanjing, 210008 Jiangsu P. R. China; Department of Medical Oncology, The Sixth Affiliated Hospital of Sun Yat-sen University, Guangzhou, 510655 Guangdong P. R. China

**Keywords:** Metastatic colorectal cancer, Maintenance therapy, Consensus

## Abstract

The impact of maintenance therapy on progression-free survival and overall survival as well as quality of life of Chinese patients with metastatic colorectal cancer has long been under discussion. Recently, some phase III clinical trials have revealed that maintenance therapy can significantly prolong the progression-free survival while maintain an acceptable safety profile. Based on this evidence and common treatment practice in China, we now generated one Expert Consensus on Maintenance Treatment for Metastatic Colorectal Cancer in China to further specify the necessity of maintenance therapy, suitable candidates for such treatment, and appropriate regimens.

## Consensus

The incidence of colorectal cancer (CRC) has increased annually. CRC is one of the five most common cancers in Chinese males and females [1]. In recent years, the outcome of metastatic colorectal cancer (mCRC) has been significantly improved due to the introduction of novel drugs and biological agents. Patients are achieving improved parameters such as increased response rate, prolonged progression-free survival (PFS), and prolonged overall survival (OS). Several clinical trials demonstrated that patients who benefited from initial chemotherapy alone or in combination with targeted agents can also benefit from maintenance therapy by having prolonged time to disease recurrence or deterioration, prolonged PFS, delayed disease progression, and improved quality of life (QoL) [2–5]. Although there are several clinical trials on maintenance therapy, none of them has provided guidance on the selection of candidates, regimens, and timing. Therefore, the present paper summarizes the evidence and expert consensus from relevant clinical trials.

## Necessity for and candidates of maintenance therapy

### Definition of maintenance therapy and its necessity

Maintenance therapy is the continued use of less potent and toxic drugs when the maximum response stabilizes after a defined period of first-line treatment, such that the patient would have a prolonged PFS, few adverse effects (AEs), delayed recurrence of tumor-associated symptoms, and, consequently, an improved QoL.

**Fig. 1:**
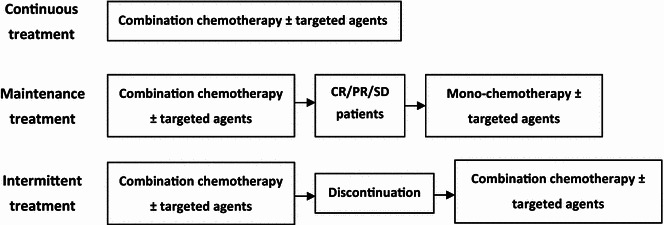
Schematic diagram of continuous, maintenance, and intermittent treatment patterns. FOLFOX [oxaliplatin, 5-fluorouracil (5-FU) plus folinic acid], FOLFIRI (irinotecan, 5-FU plus folinic acid), CapeOx (capecitabine plus oxaliplatin), and FOLFOXIRI (oxaliplatin, irinotecan, 5-FU plus folinic acid) regimens were used for combination chemotherapy. Bevacizumab or cetuximab was used as targeted agents. *CR* complete remission, *PR* partial remission, *SD* stable disease

Patients who have benefited from first-line standard chemotherapy alone or in combination with targeted treatment could have three options (Fig. 1; Table 1):

**Table 1:** First-line treatment protocols for patients with metastatic colorectal cancer (mCRC) who are able to tolerate highly intensified treatment

Chemotherapy protocol	Route and cycles of administration
FOLFOX (mFOLFOX6)^a,b^	Oxaliplatin 85 mg/m^2^, intravenous infusion (IV) for 2 h on Day 1; leucovorin (LV) 400 mg/m^2^, IV for 2 h on Day 1; 5-fluorouracil (5-FU) 400 mg/m^2^, IV on Day 1, and then 1200 mg/m^2^ continuous IV for 2 days (total dose 2400 mg/m^2^ for 46–48 h) Protocol repeated every 2 weeks
CapeOx^a^	Oxaliplatin 130 mg/m^2^, IV for >2 h on Day 1; capecitabine 850–1000 mg/m^2^, oral administration twice daily on Days 1–14, and then discontinued for 7 days Protocol repeated every 3 weeks
FOLFIRI^a,b^	Irinotecan 180 mg/m^2^, IV > 30–90 min on Day 1; LV 400 mg/m^2^, IV for 2 h with irinotecan on Day 1; 5-FU 400 mg/m^2^, IV on Day 1 and then 1200 mg/m^2^/d continuous IV for 2 days (total dose 2400 mg/m^2^ for 46–48 h) Protocol repeated every 2 weeks
FOLFOXIRI^a^	Irinotecan 165 mg/m^2^, oxaliplatin 85 mg/m^2^ plus LV 400 mg/m^2^, IV on Day 1; 5-FU 1600 mg/m^2^ per day, continuous IV for 2 days (total dose 3200 mg/m^2^ for 48 h) Protocol repeated every 2 weeks

^a^The regimen can be used alone or in combination with bevacizumab (5 mg/kg in 2-week protocol or 7.5 mg/kg in 3-week protocol, IV on Day 1 of each cycle)

^b^The regimen can be used alone or in combination with panitumumab (6 mg/kg, IV > 60 min, repeated every 2 weeks) or cetuximab (only for patients with wild-type *KRAS*/*NRAS*; initial dose 400 mg/m^2^, IV > 2 h, and then 250 mg/m^2^, IV > 60 min once a week or 500 mg/m^2^ every 2 weeks)

1. Continuous treatment: continued use of standard chemotherapy alone or in combination with targeted agents until disease progression or intolerable toxicity occurs.
2. Maintenance treatment: a standard and potent regimen with highly toxic drugs is discontinued and replaced by a less potent maintenance regimen with less toxic drugs, which usually comprises a first-line drug and another drug or targeted agent with no cross-resistance to first-line treatment. Maintenance treatment is usually used at a lower dose.
3. Intermittent treatment: complete discontinuation of chemotherapy and targeted drugs.

The prolonged use of standard chemotherapy alone or in combination with targeted agents will escalate drug toxicity. The accumulated neurotoxicity of the oxaliplatin-based protocol has contributed to the discontinuation of chemotherapy in patients who consistently responded to chemotherapy. However, these patients would have an improved outcome if they switched to maintenance treatment with less potent and toxic drugs [2]. The OPTIMOX1 study revealed that maintenance treatment was comparable to continuous treatment with regard to its efficacy, but it significantly reduced AEs and improved the patients’ QoL [6].

The results from the OPTIMOX2 study confirmed that maintenance treatment is superior to intermittent treatment in terms of clinical efficacy [7]. In the OPTIMOX2 study, patients with mCRC discontinued chemotherapy (chemotherapy-free interval group), or they were switched to a simplified maintenance treatment with 5-fluorouracil (5-FU) or leucovorin (LV) (maintenance group) following 6 cycles of modified FOLFOX7 until disease progression. The results showed that the maintenance group surpassed the chemotherapy-free interval group in OS (23.8 vs. 19.5 months, *P* = 0.042) and PFS (8.6 vs. 6.6 months, *P* = 0.0017) [7].

Therefore, compared with the other two patterns, maintenance treatment after first-line treatment is essential for patients with mCRC, and it is an appropriate therapeutic strategy for most patients.

### Candidates for maintenance treatment

The benefit is maximized after 3–6 months of first-line treatment. Patients can present complete response (CR), partial response (PR), or stable disease (SD). However, those with mCRC who cannot tolerate the toxicity of continuous combination therapy alone or in combination with targeted drugs are candidates for maintenance treatment.

The results from the OPTIMOX2 study indicated that maintenance therapy can be suitable for high-risk patients with poor prognosis, whereas intermittent therapy can be applied to low-risk patients with fair prognosis [7]. However, selecting indicators for candidates with improved outcomes at baseline levels are poorly defined; therefore, they deserve further study.

## Protocols and duration of first-line treatment

### Protocols of first-line treatment

For patients with mCRC who can tolerate highly intensified treatment, first-line recommendations include FOLFOX [oxaliplatin, 5-fluorouracil (5-FU) plus folinic acid], FOLFIRI (irinotecan, 5-FU plus folinic acid), CapeOx (capecitabine plus oxaliplatin), and FOLFOXIRI (oxaliplatin, irinotecan, 5-FU plus folinic acid) regimens (Fig. 1) [8], all of which can be used in combination with bevacizumab [8–12]. FOLFOX and FOLFIRI regimens can be combined with panitumumab or cetuximab (for patients with wild-type *KRAS*/*NRAS* only) [13].

### Duration of first-line treatment

Most patients will obtain maximum response after 2–3 months of chemotherapy, and they are required to stop standard chemotherapy due to neurotoxicity and bone marrow suppression after 4–6 months. Clinicians must decide the duration of first-line treatment on the basis of balancing efficacy and safety.

The 2014 European society for medical oncology (ESMO) Clinical Practice Guidelines for diagnosis, treatment, and follow-up of mCRC recommends a first-line treatment duration of 3–6 months [14]. Several phase III clinical trials suggested that if the patient obtains a maximum response after 3–6 months of first-line treatment, he/she can switch to maintenance therapy [5, 6, 15].

The National Comprehensive Cancer Network (NCCN) guidelines strongly recommend discontinuation of oxaliplatin after 3 months of FOLFOX or CapeOx chemotherapy and even earlier if intolerable neurotoxicity occurs. The reminder drugs in the maintenance therapy are retained until 6 months or disease progression. Patients presenting signs of neurotoxicity should not use oxaliplatin until near-total resolution of neurotoxicity.

## Protocol of maintenance treatment

### Maintenance chemotherapy

Currently, targeted agents are not affordable to many Chinese patients as a first-line treatment. For them, chemotherapy alone can be used as maintenance treatment.

In the XelQuali study, patients with unresectable mCRC who obtained stable disease (76%) after 4 cycles of first-line CapeOx treatment continued to have single-agent capecitabine maintenance [3]. The results showed that the median PFS was 6.7 months, and the median OS was 20.5 months for all recruited patients. In the capecitabine maintenance subgroup, the median PFS and OS were 8.1 and 23.1 months, and the occurrence of AEs of all grades (neuropathy, diarrhea, and lethargy) was significantly reduced. The occurrence rates of grade 1/2 and grade 3 AEs were 77% and 7% in the experimental group and were 47% and 3% in the control group. The results indicated that single capecitabine maintenance for patients obtained CR, PR, or SD after short-term CapeOx provided an effective and tolerable therapeutic option. This protocol minimized accumulated neurotoxicity by reducing repeated injections of oxaliplatin. It is more cost-effective and convenient than continuous treatment to be used for patients and healthcare providers [3].

The single-agent capecitabine maintenance protocol has been fully verified in Chinese patients with mCRC. Sun Yat-sen University Cancer Center conducted a phase II study of CapeOx as a first-line treatment followed by capecitabine maintenance in patients with mCRC. Among the 124 patients with treatment-naïve mCRC receiving a median 6 cycles of CapeOx, 62 with no disease progression chose discontinuation or continued to receive oral capecitabine (1000 mg/m^2^, twice daily) until disease progression or intolerable toxicity occurred. Among them, 22 received maintenance treatment. The patients who did or did not have maintenance treatment were similar in baseline characteristics. The results showed that at the end of the study (median follow-up, 20 months), 19 (86.3%) patients in the maintenance group were still alive (range, 8–37 months); therefore, OS could not be calculated. The median duration of disease control was superior in the maintenance group than in the non-maintenance group (14 vs. 9 months, *P* = 0.041). Among the 22 patients in the maintenance group, only 1 discontinued treatment due to grade 3 hand-foot syndrome after receiving 2 cycles of capecitabine. Four patients (18.2%) had grade 1/2 hand-foot syndrome, and the rest had a mild AE [16].

Sun Yat-sen University Cancer Center published the results of a phase III multi-center study on continuous capecitabine maintenance after CapeOx or FOLFOX in 2015 [17]. In total, 274 patients with mCRC were enrolled, and they were randomized at a 1:1 ratio into capecitabine maintenance (1000 mg/m^2^, twice daily for 14 days, then chemo-free interval for 7 days; *n* = 136) or non-maintenance observation (*n* = 138) after CapeOx or FOLFOX for 18–24 weeks. The results showed that the maintenance group surpassed the observation group in PFS (10.43 vs. 7.82 months, *P* < 0.001). Two patients (1.5%) in the maintenance group withdrew from the study due to a toxic reaction, but most patients tolerated the treatment well.

Another similar study on capecitabine maintenance after first-line treatment was performed by Peking University Oncology Hospital, with 85 recruited mCRC patients obtaining controlled disease (CR, PR, or SD) after first-line CapeOx, FOLFOX, or FOLFIRI [4]. Thirty-three patients chose to receive capecitabine maintenance, and the others were observed without maintenance. The results suggested a superior prognosis of the maintenance group compared with the non-maintenance group. The median time to progression (TTP) was 9.0 months in the maintenance group and 6.5 months in the non-maintenance group (*P* = 0.007); OS was 40.4 and 21.9 months, respectively (*P* = 0.015). Among the patients on maintenance treatment, 6 (18.2%) had grade 1 hand-foot syndrome, 1 (3.0%) had grade 3 diarrhea, and 1 (3.0%) had grade 3 thrombocytopenia. All of these events were controlled by supportive treatment and did not contribute to early withdrawal [4].

### Maintenance therapy with targeted agents

#### Maintenance with chemotherapy in combination with targeted agents

For mCRC patients responding to chemotherapy in combination with targeted agents, maintenance with less toxic chemotherapy (5-FU) and targeted agents are recommended. Among these schemes, the bevacizumab plus capecitabine regimen has the most compelling evidence.

In the sequential versus combination chemotherapy with capecitabine, irinotecan, and oxaliplatin in advanced colorectal cancer (CAIRO3) study, a Dutch phase III randomized controlled trial, mCRC patients were randomized to receive bevacizumab plus capecitabine doublet maintenance, or to be observed with no maintenance after 6 cycles of first-line CapeOx plus bevacizumab, and then they were re-inducted with CapeOx plus bevacizumab when disease progression occurred [5]. The results showed that the maintenance group was superior to the suspension group with regard to PFS, but there was no significant difference in QoL scores [5].

In the German AIO KRK 0207 trial, patients with mCRC were randomized to receive 5-FU plus bevacizumab maintenance, single-agent bevacizumab maintenance, or no maintenance after 24 weeks of 5-FU, oxaliplatin plus bevacizumab triplet therapy [15]. The results showed that the median PFS was 6.2, 4.8, and 3.6 months, respectively. To achieve a longer first TTP, 5-FU plus bevacizumab is a superior maintenance scheme [15].

#### Maintenance treatment with a single targeted drug

For patients responding to chemotherapy in combination with targeted drugs, if they cannot tolerate a less toxic chemotherapy regimen, individual targeted agent maintenance is a possible treatment option.

In the First-Line XELOX Plus Bevacizumab Followed by XELOX Plus Bevacizumab or Single-Agent Bevacizumab as Maintenance Therapy in Patients with Metastatic Colorectal Cancer study, the Phase III MACRO TTD study, AEs were significantly reduced in patients with single-agent bevacizumab compared with those with continuous chemotherapy [18]. This study assessed the efficacy on patients with mCRC who switched to single-agent bevacizumab maintenance or continued the scheme after receiving CapeOx plus bevacizumab induction. The results showed that median PFS (9.7 vs. 10.4 months), median OS (20.0 vs. 23.2 months), and response rate (49% vs. 47%) were not significantly different between the two groups. However, the rate of AEs including diarrhea, food-hand reaction, and neuropathy was significantly higher in patients continuing chemotherapy than in patients switching to maintenance treatment [18].

In the MACRO-2 study, patients received FOLFOX plus cetuximab for 8 cycles, and then they were randomized to receive single-agent cetuximab maintenance or continuous FOLFOX plus cetuximab [19]. The results revealed that the efficacy of single-agent cetuximab maintenance was not inferior to continuous combination therapy (PFS: 8.9 vs. 9.8 months, *P* = 0.09; OS: 23.6 vs. 22.2 months, *P* = 0.54; response rate: 47% vs. 39%, *P* = 0.33) [19].

The combination maintenance with two or more targeted agents presented an increased degree of AEs instead of improved benefits; therefore, epidermal growth factor receptor (EGFR)- and vascular endothelial growth factor receptor (VEGF)-targeted agent combination was not recommended.

The Panitumumab Advanced Colorectal Cancer Evaluation (PACCE) study found that for oxaliplatin- or irinotecan-based chemotherapy in combination with bevacizumab, when panitumumab was added, PFS was significantly shortened and toxicity was significantly strengthened, regardless of wild-type or *KRAS* exon 2 mutations [20]. Similar results were obtained from the CAIRO2 study by adding cetuximab to capecitabine, oxaliplatin, and bevacizumab [5]. Therefore, the community of experts is strongly averse to combining therapies that target EGFR (cetuximab or panitumumab) and VEGF (bevacizumab).

## Maintenance scheme for patients with first-line failure

There is a lack of studies on maintenance treatment for patients who had a first-line failure and began to receive second-line or higher chemotherapy alone or in combination with targeted agents. Consequently, therapeutic principles and options can be made in reference to recommendations for first-line treatment.

## Comprehensive evaluation and prospect of maintenance treatment

In summary, physicians are required to manage the complete process of mCRC by comprehensively considering the benefits for life expectancy and QoL, while also considering factors including the patient’s will, employment, life, and economy, to choose the most suitable treatment regimen. Maintenance therapy is essential for most mCRC patients. As a therapeutic strategy with less toxicity and stable efficacy, it significantly reduced AEs and improved patients’ QoL. To select the scheme of maintenance treatment, maintenance with targeted agents in addition to less toxic chemotherapy (5-FU) is recommended for mCRC patients responding to chemotherapy with a targeted treatment combination. Patients who underwent only chemotherapy as a first-line treatment can undergo less toxic chemotherapy alone. Single-agent capecitabine maintenance is the most evidence-based scheme, and it has been fully verified in Chinese patients with mCRC. Oral single-agent capecitabine maintenance is more convenient to use; thus, it has an improved patient compliance.

In addition to the therapeutic strategies described above, investigators are exploring new therapeutic options. Metronomic chemotherapy is a frequent use of low-dose chemotherapeutics. Compared with conventional large dose impact chemotherapy, metronomic chemotherapy is cytotoxic to tumors, and it can also facilitate the apoptosis of endothelial cells, suppress angiogenesis, and reduce AEs. Preliminary results of in vivo and in vitro studies suggested that metronomic chemotherapy with capecitabine chemotherapy can suppress the proliferation of tumor cells in CRC, and it is comparable to cyclophosphamide (CTX) metronomic chemotherapy with respect to efficacy [21]. In a Swedish study, patients with *KRAS* mutations who achieved CR, PR, or SD by receiving chemotherapy in combination with bevacizumab were randomized to receive a metronomic dose of bevacizumab or capecitabine (500 mg, oral administration, twice daily). The median PFS was 3.8 months in the bevacizumab group and 3.7 months in the capecitabine group [22]. Capecitabine metronomic chemotherapy is a novel scheme that merits testing. Its efficacy and safety in mCRC patient maintenance requires further study.

Many questions about maintenance therapy remain unanswered, e.g., what kind of patients can achieve the best benefits from maintenance therapy? Is there any relevant biomarker to identify them? In existing studies, follow-up treatment schedule following disease progression is also a focus of future exploration. Answers to these questions will aid the identification of the best therapeutic strategy for patients with late-stage CRC.
